# Important factors to consider when treating children with chronic fatigue syndrome/myalgic encephalomyelitis (CFS/ME): perspectives of health professionals from specialist services.

**DOI:** 10.1186/s12887-017-0799-7

**Published:** 2017-02-01

**Authors:** Roxanne M. Parslow, Alison Shaw, Kirstie L. Haywood, Esther Crawley

**Affiliations:** 1 Centre for Child and Adolescent Health, School of Social & Community Medicine, University of Bristol, Barley House, Oakfield Grove, Bristol, BS8 2BN UK; 2 Centre for Primary Care Research, School of Social & Community Medicine, University of Bristol, Canynge Hall, Bristol, BS8 2PS UK; 3 Royal College of Nursing Research Institute, School of Health and Social Studies, University of Warwick, Coventry, CV4 7AL UK

**Keywords:** Chronic fatigue syndrome/myalgic encephalomyelitis (CFS/ME), Children, Health Professionals, Qualitative

## Abstract

**Background:**

Paediatric Chronic Fatigue Syndrome (CFS)/Myalgic Encephalomyelitis (ME) is relatively common and disabling. Improving treatment requires the development of Patient Reported Outcome Measures (PROMs) that enable clinicians and researchers to collect patient-centred evidence on outcomes. Health professionals are well placed to provide clinical insight into the condition, its treatment and possible outcomes. This study aimed to understand the perspectives of specialist paediatric CFS/ME health professionals and identify outcomes that are clinically important.

**Methods:**

Focus groups and interviews were held with 15 health professionals involved in the care of children with CFS/ME from the four largest specialist paediatric CFS/ME services in the NHS in England. A range of clinical disciplines were included and experience in paediatric CFS/ME ranged from 2 months to 25 years. Ten participants (67%) were female. Focus groups and interviews were recorded, transcribed verbatim and data were analysed using thematic analysis.

**Results:**

All health professionals identified the impact of CFS/ME across multiple aspects of health. Health professionals described four areas used to assess the severity of the illness and outcome in children: 1) symptoms; 2) physical function; 3) participation (school, activities and social life); and 4) emotional wellbeing. They also described the complexity of the condition, contextual factors and considerations for treatment to help children to cope with the condition.

**Conclusions:**

Clinically important outcomes in paediatric CFS/ME involve a range of aspects of health. Health professionals consider increases in physical function yet maintaining school functioning and participation more widely as important outcomes from treatment. The results are similar to those described by children in a recent study and will be combined to develop a new child-specific PROM that has strong clinical utility and patient relevance.

## Background

Chronic Fatigue Syndrome/Myalgic Encephalomyelitis (CFS/ME) is a complex condition that includes a range of symptoms such as debilitating fatigue, sleep disturbances, pain, cognitive dysfunction, headaches, dizziness and sweats. Symptoms vary between individuals and fluctuate in intensity and severity [1]. Paediatric CFS/ME is relatively common with a prevalence of between 0.4% and 2.4% [2–5] in population studies and between 0.06% and–0.1% [6, 7] in studies based in hospital settings. It is increasingly recognised as an important disabling condition [1, 8, 9]. Physical activity is usually limited and loss of schooling occurs, ranging from low attendance to extended periods of absence [10–12].

There is little research on effective treatments [13]. NICE recommends that children with CFS/ME should be offered either Cognitive Behavioural Therapy (CBT), Graded Exercise Therapy (GET) or activity management [1]. In a condition with no objective measures of outcome, it is important to collect subjective measures of health-related quality of life (HRQoL) directly from patients. Improving the evidence base requires the development of questionnaires or Patient Reported Outcome Measures (PROMs) that enable clinicians and researchers to collect patient-centred evidence on HRQoL outcomes for these treatment approaches. PROMs can also be used in shared decision making [14–16] to prompt discussion between patients and health professionals, alert professional to the patient’s concerns about their HRQoL and clarify the patient’s priorities for care [17]. The utility of PROMs is dependent on the relevance and acceptability of the PROM to both patients and clinicians. There is currently a lack of well-developed PROMs in CFS/ME [18, 19].

We have recently described a children-derived conceptual model which describes the impact and outcomes of CFS/ME and will be the basis of a new child-specific CFS/ME PROM [20]. Clinicians need to be involved in the development of a PROM alongside children if it is to be used clinically and in trials [21–23]. Clinicians are aware of how outcomes manifest across a wide range of people and contexts [24]. Optimal content validity in PROM development includes both perspectives where appropriate, rather than prioritizing one over the other [24]. Children with CFS/ME receive specialist care from a range of clinical disciplines: paediatricians, nurses, physiotherapists and psychologists who have accumulated knowledge and experience of the condition. However, little is known about how specialist CFS/ME clinicians view the condition.

Professionals working with adults with CFS/ME have described the complexity of the condition and the onus on the person with CFS/ME to manage their illness [25]. A recent study found professionals working with children with CFS/ME in general paediatric clinics, ‘work with uncertainty’ and use previous experiences to inform the labels they give to children [26]. However, they did not explore management of the condition or outcomes of importance. This study aimed to understand the perspectives of specialist paediatric CFS/ME health professionals and identify the outcomes that are clinically important.

## Methods

The study sought to explore the views and experiences of health professionals who work in specialist paediatric CFS/ME services in England and have regular contact with children with CFS/ME. Qualitative methods were used.

### Setting

We purposefully sampled specialist CFS/ME paediatric services within the NHS. The four largest specialist services were recruited, based in the following UK regions: South West, London, East of England and the North East. The health professionals treated children (<19 years old). Two of the clinicians only treated teenagers. The services provided activity management, CBT and GET as treatment approaches.

### Participants

Within the sampled specialist CFS/ME settings, we planned to sample health professionals from a range of professional backgrounds, who had regular contact with children with CFS/ME. Lead clinicians from the specialist services were approached by email and asked to cascade the information to eligible multidisciplinary clinical team members. While we sought to be purposeful in participant selection, our sampling relied on access via a gatekeeper (the lead clinician) and therefore, our final sample was a convenience sample of those clinicians who responded.

### Data collection

Data were collected using a mixture of focus groups, paired and individual interviews, as determined by participant preference and practicality. Where possible, focus groups were conducted at the health professionals’ hospital premises to facilitate group interaction and breadth of discussion [27]. However, if focus groups were not practically feasible or at participants’ request, interviews were conducted face to face in private rooms within the hospital or by telephone. Interviews were either individual (with a single participant) or paired (with two participants). Paired or dyadic interviews sit somewhere between a group and individual interview, allowing relatively detailed contributions from individual participants while also giving opportunities for comments from one participant to prompt responses from the other [28]. We acknowledge that these different forms of data collection can generate different forms of qualitative data. In the context of this study, each were appropriate for generating data relevant to our study aim and were offered flexibly as a practical strategy for facilitating participant engagement. One author (RP) facilitated the focus groups and conducted all interviews.

A flexible topic guide was developed following discussions with all authors to enable participants to talk about their views and to raise issues of importance, but also to provide some consistency between the different groups and interviews (Appendix A). The guide covered: 1) the service context within which the professional(s) worked; 2) current use of PROMs and 3) views about the aspects of health that are important in their assessment of outcomes and shared decision-making with children with CFS/ME. The topic guide was revised as the study progressed to reflect emerging issues raised during the focus groups and interviews.

### Data analysis

Analysis was led by RP. Preliminary analysis was conducted alongside data collection, to enable data gathered earlier on to inform subsequent data collection. Data were transcribed verbatim and checked for accuracy. Notes made after each focus group and interview were considered alongside relevant transcripts. Data were analysed thematically [29], incorporating a mixture of deductive and inductive coding, to enable development of both anticipated and emergent themes. Transcripts were read line-by-line for content and meaning, and provisional codes were applied to relevant sections of text. Coding was undertaken using the software package NVivo 10 [30]. This process led to the development of an initial coding framework. Other members of the research team (EC, AS, KH) read and independently coded a sub-set of the data to incorporate different perspectives and enhance interpretation. The coding framework was refined, with new codes added and existing codes merged or split. Through this process, broader categories and higher-level recurring themes were developed. Data within themes were examined for disconfirming and confirming perspectives. Finally, a narrative summary of the findings was written, integrating illustrative data and giving attention to the different perspectives represented. The research team included a range of disciplines, both clinical and methodological (qualitative methods, PROMS). In presenting findings, data have been anonymised to protect confidentiality.

## Results

Eighteen health professionals were approached via email. Fifteen health professionals consented to participate. Three did not participate due to lack of time. A range of clinical disciplines were included (Table 1) and experience in paediatric CFS/ME ranged from 2 months to 25 years. Ten participants (67%) were female. Two focus groups (compromising 5 participants in one and 4 in the other), a paired interview, two individual face-to-face interviews and two telephone interviews took place. These were conducted at participants’ place of work or over the telephone and lasted between 43 and 61 min (median 52 min).

**Table 1:** Demographic Profile of Participants

Participant I.D	Gender	Professional Group	Years in Paediatric CFS/ME	Focus Group/Interview
HP1	Male	Medicine	2	Focus Group
HP2	Male	Health Psychology	6	Focus Group
HP3	Female	Physiotherapy	22	Focus Group
HP4	Female	Physiotherapy	2	Focus Group
HP5	Female	Nursing	2 months	Focus Group
HP6	Female	Nursing	15	Focus Group
HP7	Male	Paediatrics	7	Focus Group
HP8	Female	Clinical Psychology	3	Focus Group
HP9	Female	Physiotherapy	3	Focus Group
HP10	Female	Clinical Psychology	10	Paired Interview
HP11	Female	Clinical Psychology	2	Paired Interview
HP12	Male	Paediatrics	11	Interview
HP13	Female	Occupational Therapy	9	Interview
HP14	Female	Paediatrics	6	Telephone Interview
HP15	Male	Medicine	25	Telephone Interview

All health professionals talked about the complexity of paediatric CFS/ME and the impact across multiple aspects of health. They also described the impact of context and strategies they adopted to help children to cope with the condition.

## Important health outcomes

Health professionals described four areas that they currently use to assess the severity of illness and outcome in children with CFS/ME: 1) symptoms; 2) physical function; 3) participation (school, activities and social life); and 4) emotional wellbeing.

### Symptoms

Health professionals described the wide range of symptoms children with CFS/ME present with which limit their ability to do things.

*“… it’s a complex area. So there’s the physical side of it, obviously, so the fatigue the poor sleep, um, lots of children with stomach problems, um, lots of children with cognitive problems” (HP14)*

A reduction in symptoms or a child’s ability to manage them better was felt to be an important outcome. Improving the quality of sleep was viewed by all participants as an essential outcome, as sleep impacts a child’s ability to manage other areas. Other symptoms were not as prevalent (e.g. loss of appetite), but were important to detect and treat.

*“…diminishing of symptoms, either a decrease in symptoms or ability to manage them better…” (HP10)*

*“Anchoring their sleep is absolutely fundamental to getting better, so that’s absolutely really important” (HP1)*

*“…loss of appetite become a norm, but actually they haven’t mentioned it because it’s not predominant along with maybe fatigue, pain…” (HP12)*

### Physical function

Health professionals were additionally concerned about the impact of symptoms on a child’s ability to do daily physical activities. They described different levels of physical function with some children: unable to do anything, walking on crutches or being physically exhausted after a full school day. In some cases, health professionals felt reducing “payback”, or the increase in symptom severity limiting physical function after doing more activity, important to improve and measure.

*”…they say they are feeling or unable to do anything, we tend to sort of, respond to that.” (HP8)*

*“We’ve had some who have not been able to walk, or walk on crutches.” (HP13)*

*“If they say, “Well, I still don’t want to get out of bed, but it’s finding it easier,” that’s quite a good mark of disease improvement. And the same is if you’re doing a bit more physical activity without it being absolutely exhausting, and you’re not getting payback the next day.” (HP1)*

### Participation: school, leisure activities and social life

Social withdrawal was described as a key consequence of CFS/ME. Health professionals considered both school and social or leisure activities as important signs of participation. They described one overall health outcome domain of participation where school and leisure activities such as sport and hobbies provide children with the ability to participate in normal social structures, with a cumulative impact for improvement.

*“So your social participation with your friends might be when you go and play football.” (HP7)*

Most health professionals felt increasing school attendance suggested an improvement in health. However, some thought that school attendance could be a misleading outcome as it is often reduced during treatment and does not necessarily reflect a child's disability. Children with CFS/ME may put all their energy into school leaving them exhausted.

*“…whether they’re getting worse or not, or whether they’re improving, is whether they are able to increase the amount they’re going to school” (HP13)*

*“…in a school situation, or a social situation, the young person will give 110% to appear normal, and will use up all their energy …But when they get home, …They’re completely wiped out.” (HP3)*

*“…I perversely think sometimes less school attendance may be a sign of improvement.” (HP12)*

### Emotional wellbeing

Emotional wellbeing emerged as a significant outcome to monitor and address, with some health professionals proposing that it is more important than school attendance. Health professionals described low mood and frustration in children, caused by being unable to do things. School stress and anxiety about returning to school were additional dimensions. They felt that this low mood, anxiety and stress can develop into formal anxiety and depression.

*“Emotional wellbeing is I think is what I’d say, in school or out of school.” (HP7)*

*“Yes; they’re just frustrated that they can’t do stuff, in the moment, that they want to do.” (HP1)*

*“some of them become clinically depressed as a result of their illness, and that needs to be recognised and treated.” (HP15)*

Social anxiety was raised as a particular problem in children with this condition. Children were said to be anxious about being able to cope with their symptoms in social situations. Lack of understanding from others played a part with problems with reintegration into schools and leisure.

*“It’s often because they’re worried, they have got so (..) affected by CFS and also frightened by the symptoms they are experiencing,…so become frightened of the idea of trying to retain or re-attain normality” (HP8)*

### Self esteem

Health professionals described the ‘loss’ felt by children due to what they could no longer do as a consequence of having CFS/ME. Not being able to fulfil their goals had a role to play in how they saw themselves. Health professionals often provided examples of athletic children who were no longer able to perform their sports or academic children who were unable keep up with schooling impacting their self-esteem.

*“there are quite a few children we’ve had who been, um, swimmers and athletes, and that’s been the thing that has given them their self-confidence and self-esteem, and their sense of, you know value, and they’ve had to stop doing that, and that’s really difficult.” (HP13)*

*“…the ones particularly at school who feel like failures all the time, because they’re not reaching their potential or their goals…” (HP1)*

### Developmental differences in important outcomes

The impact of the condition and important outcomes to children identified by health professionals was felt to vary developmentally. Health professionals reported younger compared to older children concerned with being ‘normal’ and ‘back doing what their friends are doing’.

*“…it varies by developmental and time factors” (HP8)*

*“They tend to want to be back at school seeing friends” (HP8)*

Teenagers face extra complexities such as the disruption of natural independence from parents as well as spending time with a boyfriend/girlfriend. Health professionals described more issues around school and academic achievement in adolescents; problems with memory and concentration impacted children’s ability to keep up with school work. They talked about stress due to exams and falling behind. Health professionals felt there was higher susceptibility for anxiety and depression at this stage.

*“…especially with teenagers, sort of, natural break where they’re becoming more independent from their parents is being interrupted” (HP13)*

*“We tend to get a lot with extra issues like anxiety and stress and worrying and low mood coming in then because they have stress for exams.”(HP8)*

At a pivotal developmental stage where many young people are completing important exams, having CFS/ME can demotivate them and make them anxious about their future. Older children were said to have particular worries about the future and achieving their goals.

*“as they get older, there’s so much more around, having to drop GCSEs or ‘how am I going to manage my A Levels’, or ‘what am I going to do if I want to go to university, how is this going to impact on me’ and those kind of real future worries” (HP11)*

## Complexity, context and facilitators to coping

Health professionals described the difficulty of treating children with CFS/ME due to the variability and fluctuation of the condition and environmental barriers preventing children from returning to normal. A number of strategies were employed to help children cope with the condition.

### Complexity and circularity in CFS/ME

Health professionals talked about the complexity of the condition with symptoms varying between children.

*“I mean, in terms of sleep problems…either slept huge number of hours, which is like sort of 18 out of 24 h, or we’ve had one particular young person who slept two hours a night for about two years…So it’s really variable, and that’s really difficult to deal with.” (HP14)*

Circularity was also described as a feature of the condition. Children experience a ‘boom and bust’ pattern with increased symptom severity (payback) following activity which can lead to a downward spiral of reduced activity. They additionally described the circularity of low mood as maintenance factor preventing improvement in CFS/ME. Children can have low mood due to symptoms and a lack of participation and can then become more vigilant to symptoms. This can then lower their thresholds for participation, further lowering their mood in a negative cycle.

*“Um, trying to stop that boom and bust pattern. A lot of them are still really, really pushing themselves” (HP13)*

*“Obviously, if they’ve got symptoms and that stops them participating, their emotional wellbeing deteriorates and that makes their symptoms worse, so you actually get a negative feedback loop.” (HP15)*

*“…when children are, or anyone is low or anxious, you become, quite um, hypervigillant to what is going on in your body” (HP11)*

### Facilitators to help children cope

Flexible strategies were required to treat the variable severity of symptoms and functional ability of individual children.

*“If the child is saying, “I’m in pain and actually it’s only when I’m tired or at the end of the day,” that’s different to a child says, “Well I wake up and I am in pain all the time,” and different strategies for management.” (HP12)*

Considering the individual functional level and priorities of children when setting treatment goals was highlighted. Health professionals described how they could be working with an athletic child one minute and then a child who only wants to see their friends the next. Or a child that wants to return to extracurricular dance versus a child who just wants to be able to wash their hair.

*“…they might attend loads of school but all of their efforts are into attending school and they might see no friends and do nothing outside with the family.” (HP11)*

*“… she had an objective, and it was very small, but I think that’s really important,… “I’d like to wash my hair,” because getting upstairs where the shower is, such an effort.” (HP13)*

Health professionals described how, in some cases, children appeared to be improving in terms of function whilst symptoms remained the same. Therefore, coping and the ability to do things despite symptoms was an important focus for health professionals. Health professionals concentrated on activity management and setting baselines to reduce boom and bust patterns and increase children’s physical function and their ability to do things. They recognised the importance of school for academic, social and emotional development and therefore the delicate balance of reducing school to improve functioning, yet maintaining a sense of normality and contact for children.

*“It’s quite interesting because they will be coping and managing and doing more and more and more but still complaining that they feel absolutely exhausted. But the reality is they’re not crashing anymore and their concentration is returning and all those other things.” (HP10)*

*“…if they are getting into school more and more but their wellness score is staying the same or going down, I would be concerned and talk with them about decreasing the amount of school they do.” (HP6)*

*“…you also look at what they’re doing outside socially as well, so ask people about social contact, friends, do you go out, what do you do at weekends?” (HP12)*

All health professionals in this study worked in a multidisciplinary team and integrated psychological approaches in order to manage negative cycles of low mood. There was a need to give children back the value they had lost and rebuild their self-esteem; refocusing on realistic goals and focusing positively on what can be done. Several health professionals talked about providing hope for the future, particularly for older children looking at alternative pathways for children to achieve their goals. They often used the success of previous patients to encourage and support those at an earlier stage who don’t see a future or even see themselves getting better.

*“…CBT has a really important role to deal with some of the being negative, um, views about the illness, and about symptom management and moving people from ‘I’ve got pain and it’s difficult, therefore you know I can’t do anything about it, therefore my pain is worse’, trying to break those cycles as well.” (HP12)*

*“I’m much more interested in getting them to focus on what they can do.” (HP15)*

*“I think one of the most important things about our service is giving those young people hope” (HP13)*

### Contextual factors

Health professionals identified external environmental factors that can act as a barrier to children with CFS/ME returning to normality. These included understanding, attitudes and support from others (friends, school and family). Due to a lack of understanding from the community, children with CFS/ME can be faced with negative attitudes and comments. Many health professionals reported that some children can become socially withdrawn.

*“because actually, friends don’t understand, and they don’t want to be seen to be different. So actually avoid social contact, so isolation, um, becomes an issue.” (HP12)*

All health professionals reported the profound impact CFS/ME has on the family. They felt this could affect the ability of families to follow clinical advice. CFS/ME has an impact on family activities and holidays, dependence on parents, parental tension regarding management of the illness, impact on siblings and the burden and cycles of guilt within the family.

*“Yeah, I’ve had a few, um, young people, um, expressing guilt that they feel that their illness is taking up a lot of their parents’ time, and is affecting the family, so they can’t go out and do the normal things that families would do, so going out on holidays or going out” (HP13)*

*“Just sort of family dynamics, or family tension, or other sorts of external stresses, erm, that are causing them to struggle to follow our advice” (HP4)*

A lack of school support can hinder reintegration. Some health professional described how returning to school can be difficult for children due to the ‘hustle and bustle’. Children can develop anxiety about their ability to cope. How supportive schools were affected children’s desire to return to school. All health professionals reported schools to vary in experience of CFS/ME, attitudes and support. Schools often have a lack of understanding about the condition and unrealistic expectations of what the child can do.

*“…it’s a big red flag for prognosis if you have an unsupportive school.” (HP1)*

*“schools really vary as to how sympathetic they are. Um, we have had a few young people who have developed sort of anxiety about going into school” (HP13)*

### Working with schools

Working with schools was a core part of treatment for all services involved in this study; educating schools, correcting unrealistic expectations and formulating reduced timetables. One health professional serving 16–18 year olds only, actively encouraged children with CFS/ME to leave mainstream school to colleges where he felt they were much more able to handle people with disabilities.

*“…schools also have unrealistic expectations of what they can do as well. It’s about educating them about what they can do” (HP15)*

*“…we encourage them to leave mainstream school and go into colleges and further education, they are much better at handling people with needs” (HP15)*

## Discussion

Health professionals working in specialist paediatric CFS/ME services describe how CFS/ME affects multiple areas of health. The variability of the condition, shrouded by social misunderstanding along with normal developmental challenges in children need to be taken into account when devising treatment programmes and understanding outcomes in children.

### Strength and limitations

To the authors’ knowledge this is the second qualitative study of health professionals working with children with CFS/ME but the first to focus on outcome. We interviewed specialist clinicians from a range of disciplines and geographical locations. The findings can inform those setting up new specialist services in paediatric CFS/ME. The impact of CFS/ME across physical, social and psychological areas of health was consistent across focus groups and interviews. The use of focus groups allowed for more debate surrounding the most important outcomes and the expression of divergent and shared perspectives within a clinical team [27]. We explored interactions in the groups (conflicts and affirmations) and these were useful to confirm the problems with focusing on school attendance as the primary outcome domain with some clinicians talking about the importance of emotional wellbeing in or out of school. It is possible that interviewing within a clinical team may have prevented junior members from speaking out, but we did not observe this happening. Where focus groups were not possible, individual interviews allowed more detailed examination of an individual’s experience and perspective. Paired interviews sit between a group and individual interview, allowing some depth while still enabling expression of shared or divergent views between clinical colleagues [28]. The paired interview included two clinical psychologists who talked more in depth about the loss experienced by children and the importance of providing hope. However, participants were not rigid in their perspectives based on their profession, for example a paediatrician talked about emotional wellbeing as the most important outcome. The findings from the different methods were similar in terms of content and range of issues discussed, even if there were variations in depth and richness of the accounts, and diversity in the roles and interactions of the researcher and participants [28, 31].

### Results in the context of previous literature

This study extends the qualitative literature on clinical perspectives of treating paediatric CFS/ME. A recent meta-synthesis of qualitative studies of health professionals treating adults with CFS/ME [32] identified several barriers to the diagnosis and management of CFS/ME, but did not explore important areas of health in treatment and assessment of outcome. A recent qualitative study on the perspectives of health professionals treating children with CFS/ME [26] explored how professionals conceptualise CFS/ME in diagnosis but did not explore what is important in treatment. In this study, clinicians discussed the need to balance treatment and outcomes across physical, social and psychological areas of a child’s life. This is consistent with the literature on the impact of CFS/ME on children’s function [10], schooling [4, 12, 33, 34], social activities [35–38], family [39–41] and emotional function [37, 38, 42–47].

The wide-ranging symptoms, physical function and individual and developmental differences between children were described by health professionals as important considerations for treatment. The complexity, co-morbid mood disorders and developmental issues adds strength to an individualised approach. This is consistent with Knight et al. [48] who identified a high number of complex and interacting symptoms in children with CFS/ME and the need for multifaceted treatment. Health professionals in this study worked with children on shared realistic goals for treatment. Health professionals have successfully managed CFS/ME in adults by taking a collaborative approach to management [32]. Goal attainment has been found to be significant predictor of quality of life improvement for people with CFS/ME [49].

Health professionals in this study described the difficulty of reducing a child’s school attendance to improve function yet maintaining participation for the child. School has been described as one of the most important outcomes for children with CFS/ME [20] and is a critical protective factor for many adverse outcomes among children and adolescents [50]. Schools were reported to vary in their understanding and support, acting as a barrier to children with CFS/ME returning to normality [51]. Working with schools was a key facilitator described by health professionals to reintegrate children and reduce social withdrawal.

Health professionals in this study described negative cycles of low mood in children. This is consistent with the strongest finding in a recent review, with higher rates psychiatric co-morbidity in children with CFS/ME compared to healthy controls or other illness groups [52]. CFS/ME families have been found to identify with the concept of vicious cycles arising as a consequence of the condition [39]. Patients are said to avoid activity due to the resulting symptoms that then leads to more symptoms due to physical deconditioning [53, 54]. In this study, setting baselines to reduce boom and bust, realistic individual goals and giving children hope for the future were key treatment priorities. Mackenzie and Wray [39] advocated the importance reassuring patients and their carers that they will recover and go on to achieve academic qualifications.

‘Symptoms’, ‘physical function’, ‘participation’ and emotional wellbeing’ described by health professionals in this study overlap with those talked about by children with CFS/ME in a recent study [20]. However, health professionals identified differences in important outcomes depending on age such as relationships with boyfriend/girlfriend(s), independence from parents and emotional difficulties in adolescents. They also identified differences in outcomes depending on the severity of CFS/ME. This could be because the child study interviewed children who were slightly younger and mild to moderately affected. This has implications for the development of a new PROM across a wide age range of children and adolescents. Differences in outcomes for those children who are severely affected by CFS/ME warrants further research.

## Conclusion

Identifying ways to increase physical function yet decrease the impact of CFS/ME on school functioning and participation more widely is a priority for health professionals. Working with schools is key to this process. Health professionals use symptoms, physical function, social participation and emotional domains to clinically understand the impact of CFS/ME on children and changes from treatment. Most of these outcomes are not currently measured in PROMs used in paediatric CFS/ME [18] and should be included in a new paediatric CFS/ME PROM. This adds to previous research which captures the perspectives of children and their parents on the experience of symptoms, outcomes and disability in the construction of a new PROM for CFS/ME that has strong clinical utility and patient relevance.

## Appendix

### Health professional interview topic guide

**Part A: Information about Current Service.**

Tell me about your service and your role within in it.

Tell me about any questionnaires you currently use with children in your service.

To what extent do you feel these are useful?

What works well with the current questionnaires in terms of capturing important outcomes?

What works less well with the current questionnaires in terms of capturing important outcomes?

**Part B: Important Aspects of Health in Paediatric CFS/ME.**

In what ways does CFS/ME affect children?

What are children diagnosed with CFS/ME typically unable to do?

How do you know if they are doing better?

How do you know if they are doing worse?

Which outcomes from healthcare do you think really matter to children with CFS/ME?

Which outcomes really matter to you when making clinical decisions about healthcare or social care for these children?

**Part C: Model of Living with Paediatric CFS/ME.**

The Health Professional will be presented with a child’s conceptual model of living with CFS/ME.

How well do you think that the proposed model captures what really matters to children with CFS/ME?

How well does the model capture what you think is important – and necessary for your clinical decision-making?

Do you think that there are any important outcomes that are missing from the model?

How do you think contextual factors impacts the experience of CFS/ME?

To what extent would you use contextual factors to influence your clinical decision-making?

**Part D: CFS/ME Paediatric Questionnaire.**

In your opinion, how might fluctuating symptoms impact the completion of paediatric CFS/ME questionnaires?

When do you feel is the best time for children to complete a questionnaire?

What do you feel should be the recall period for paediatric questionnaires in CFS/ME?

How often do you feel questionnaires should be administered?

How long do you think questionnaires should be administered for in children with CFS/ME?

What do you think is the best method to administer questionnaires?

Prompts: During the interview the researcher may use prompts to explore certain aspects in more detail.

## Abbreviations

CBT: Cognitive Behavioural Therapy
CFS: Chronic fatigue syndrome
GET: Graded Exercise Therapy
HRQoL: Health Related Quality of Life
ME: Myalgic encephalomyelitis
PROM: Patient reported outcome measure
